# The pharyngeal carriage of *Haemophilus influenzae* among healthy population in China: a systematic review and meta-analysis

**DOI:** 10.1186/s12879-019-4195-9

**Published:** 2019-06-21

**Authors:** Peng Yang, Jieming Zhang, Anlin Peng

**Affiliations:** 1 Shantou University Medical College, 22 Xinling Road, Shantou, 515041 China; 2 Wuhan University School of Health Sciences, 115 Donghu Road, Wuhan, 430071 China; 3 Wuhan Third Hospital-Tongren Hospital of Wuhan University, 241 Pengliuyang Road, Wuhan, 430061 China

**Keywords:** *Haemophilus influenzae*, Carriage, Healthy population, Meta-analysis

## Abstract

**Background:**

A nationwide investigation on the carriage proportion of *H. influenzae* among healthy populations is lacking in China. The purpose of the study was to review the prevalence of pharyngeal carriage of *H. influenzae* among healthy populations in China, and explore its influencing factors. The serotypes distribution of *H. influenzae* was also analyzed.

**Methods:**

A systematic search was conducted with key words “*Haemophilus influenzae*”, “Carriage”, and “China” or “Chinese” from inception to March 2018. After careful screening, the data of included articles were extracted with a pre-designed excel form. Then, the pooled carriage proportion of *H. influenzae* was calculated using the random effect model.

**Results:**

A total of 42 studies with 17,388 participants were included. The overall pooled carriage proportion of *H. influenzae* was 0.17 (95% *CI*: 0.13–0.21), and the carriage proportion largely varied by province. Subgroup analysis indicated that the pooled carriage proportion was 0.17 (0.13–0.21) for children, and 0.14 (0.7–0.23) for adults. There were no statistically significant heterogeneity between subgroups by age (*p* = 0.65), sex (*p* = 0.88), and season (*p* = 0.10). The pooled carriage proportion of Hib was 0.01 (0–0.02), while the carriage proportion of NTHi was 0.22 (0.13–0.31).

**Conclusion:**

In China, the carriage proportion of *H. influenzae* among healthy population was low, but it largely varied by provinces.

**Electronic supplementary material:**

The online version of this article (10.1186/s12879-019-4195-9) contains supplementary material, which is available to authorized users.

## Background

*Haemophilus influenzae* is a gram-negative coccobacilli including encapsulated strains and unencapsulated strains (nontypeable *H. influenzae*, NTHi). NTHi is an important pathogen among children that causes otitis media, sinusitis, conjunctivitis, and pneumonia, while in adults, it causes respiratory tract infection primarily in patients with chronic obstructive pulmonary disease [1]. Based on the capsular polysaccharide, the encapsulated strains can be separated into 6 serotypes (Hia-f). Hib is a major cause of bacterial meningitis among young children worldwide, before the widespread introduction of Hib vaccines [2]. The colonization of *H. influenzae* on the upper respiratory tract is a risk factor causing related diseases [3]. Thus, investigating the pharyngeal carriage of *H. influenzae* among healthy population have potential implications for public health policy.

In China, up to now, many studies investigated the pharyngeal carriage of *H. influenzae* among healthy populations. For example, Pan and colleagues [4] collected and examined the nasal swabs of 1088 children in Chaoshan region and found that 20 children (0.018) with the colonization of *H. influenzae*. Zhao and colleagues [5] tested the nasopharyngeal swabs of 472 children aged 2–5 years old in Beijing and found that the carriage proportion of *H. influenzae* was 0.095. All of these studies were conducted in specific regions and the results of these studies substantially varied, given the vast territory and population of China. The nationwide investigation on the carriage of *H. influenzae* among healthy people is lacking.

Thus, it is necessary to conduct a systematic review, to clarify the prevalence of *H. influenzae* nationally and explore the possible determinants of the prevalence of *H. influenzae*, based on the current evidences available. The present study systematically searched and reviewed the articles regarding the pharyngeal carriage of *H. influenzae* among healthy populations in China. This will help to describe the prevalence of *H. influenzae* and its influencing factors, and contribute to the decisions of public health policy.

## Methods

### Literature search and screening

Four databases (PubMed, CNKI, CBM, and Wanfang) were systematically searched with key words “*Haemophilus influenzae*”, “Carriage”, and “China” or “Chinese” from inception to March 2018, in order to collect and identify the studies regarding the carriage of *H. influenzae* among the Chinese population. In addition, the reference lists of selected studies were checked to ensure complete coverage.

The screening of studies was conducted independently by two authors. When there was the disagreement, the two authors discussed the case and came to consensus. The searched studies were imported to the NoteExpress for duplicate checking. After duplicate studies were deleted, the first round screening was conducted based on the title and abstract to excluded irrelevant studies. Then, the full text of remained studies were retrieved for the second round screening based on the following inclusion criteria: (1) observational studies carried out among healthy (without upper respiratory infection) Chinese population residing in mainland China; (2) specimens from nasopharyngeal or oropharyngeal swab; (3) culture-based inspection method of *H. influenzae* including X + V factor test, PCR, grouping serum, and so on; (4) sufficient information for the calculation of carriage proportion of *H. influenzae*. Studies only reported the proportion of carriage for single serotype of *H. influenzae* were excluded. In addition, studies from Hongkong, Macao, and Taiwan were also excluded because of the different immunization programs with mainland. Only the studies published in Chinese and English were included.

### Data extraction and quality assessment

The data extraction and quality assessment were also independently extracted by two authors, and consensus was reached by group discussion. The following data were extracted from selected articles: author, publication year, period and region of study, the number and characteristic of participants, inspection method of *H. influenzae*, and number of participants with *H. influenzae*. Moreover, the number of participants with the non-capsules type and six serotypes of *H. influenzae* were also extracted. The risk of bias of selected studies were assessed according to a guideline which was adopted from the Strengthening the Reporting of Observational Studies in Epidemiology (STROBE) [6] and included five core items: sample population, sample size, participation rate, outcome assessment, and analytical method to control for bias.

### Statistical analysis and graphing

The pooled carriage proportion of *H. influenzae* with its 95% confidence intervals were calculated by using the random effect model. Since the carriage proportion of *H. influenzae* in multiple studies less than 0.2, the data was converted with the double arcsine method before combination to ensure the 95% confidence intervals of carriage proportion for all included studies belong 0–1. Forest plots were generated to describe the carriage proportion and corresponding 95% confidence intervals (CI) for each studies and overall estimate. The Q test was used to test the heterogeneity, and *I*^*2*^ statistic was calculated to quantificationally evaluate the heterogeneity (low: 25–50%, moderate: 50–75%, and high: > 75%). To discover potential sources of heterogeneity, subgroup analyses were conducted by age, gender, season, and serotype. To assess the stability of the pooled results, sensitivity analysis was conducted by excluding each study at a time. A funnel plot was generated to visually assess the publication bias, and Egger’s test and Begg’s test were also conducted. The pooled carriage proportion of *H. influenzae* by provinces were calculated, and then a statistical map was generated using an online website for mapmaking (http://c.dituhui.com). All above analyses were conducted in the STATA 13.0 software and the Microsoft Office Excel 2013. The *p* value ≤0.05 was considered as statistically significant. All the generated pictures were properly modified by using the Adobe Illustrator CS6 software for a better visual effect.

## Results

### Search results and characteristics of included studies

**Fig. 1:**
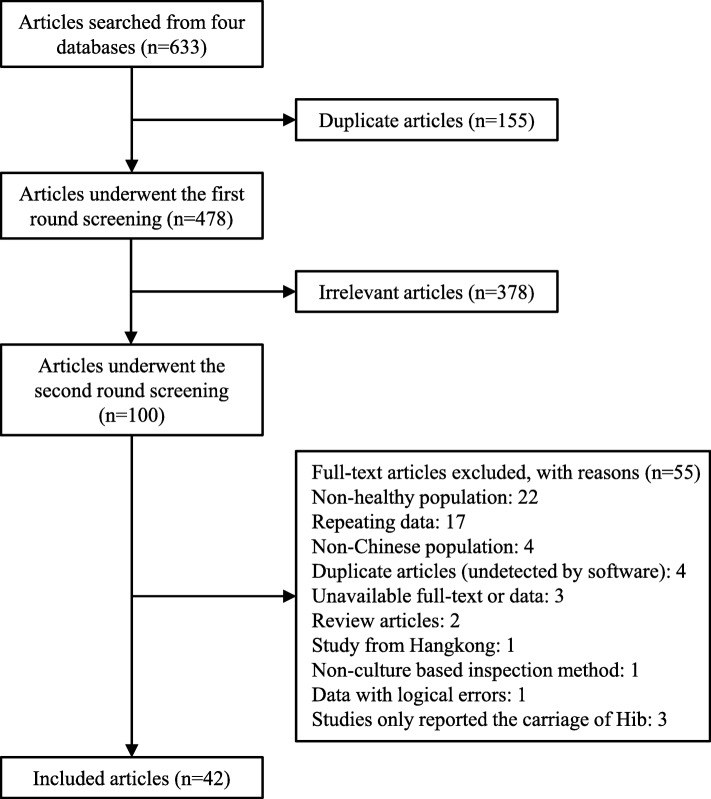
Flow chart of articles screening

A total of 633 articles were found from aforementioned four databases after the systematic search. 155 duplicate articles were excluded by using the NoteExpress software, and another 378 articles were excluded in the first round screening. Then, the full text of remaining 100 articles were reviewed. Eventually, 42 articles [4, 5, 7–46] with 17,388 participants were included (Fig. 1). No additional articles were identified from the reference lists of included articles.

The main characteristics and results of quality assessment of all included studies were listed in Table 1. The study period of all articles ranged from 1989 to 2013, and the study region covered 11 out of Chinese 31 province-level administrative regions (Excluding studies from Hongkong, Macao, and Taiwan for aforementioned reason).

**Table 1:** The characteristics and results of quality assessment of the included studies

Literature	Period	Region	Sample size	Age of subjects	Inspection methods	Participants with Hi	Quality assessment
Liu Q, 1993 [7]	1989–1990	Beijing	115	2~6y	Culture, Hi grouping serum	37	7
Lai G, 1997 [8]	1996.01	Shanghai	181	2~11y	Culture	49	6
Wang G, 1999 [9]	/	Shanghai	255	≤18y	Culture, Hi grouping serum	66	6
Zhang Y, 2000 [10]	1999	Zhangjiakou; Hebei province	319	4~60y	Culture	73	7
Chen L, 2000 [11]	/	Wuhan; Hubei province	52	/	Culture	6	6
Zhang H, 2001 [12]	1998–1999	Fuzhou; Fujian province	2053	3~6y	Culture	403	7
Cao G, 2002 [13]	2000–2001	Hangzhou; Zhejiang province	897	4~7y	Culture	159	7
Chen Q, 2002 [14]	1998–2001	Nanchang; Jiangxi province	60	1 m-14y	Culture	1	6
Hou A, 2002 [15]	1995–2000	Beijing	307	≤13y	Culture	20	7
Luo X, 2002 [16]	/	Zhongshan; Guangdong province	178	18-56y	Culture	46	6
Chen Z, 2002 [17]	/	Hangzhou; Zhejiang province	37	25.8 ± 9.8 m	Culture, PCR	3	6
Zhou H, 2003 [18]	2001–2001	Guangzhou; Guangdong province	150	3~5y	Culture	37	6
Zeng Y, 2003 [19]	/	Chongqing	400	≤5y	Culture	64	6
Hua C, 2005 [20]	2002–2003	Hangzhou; Zhejiang province	848	2~5y	Culture, Hi grouping serum	217	6
Zhao X, 2005 [21]	2002	Zhongshan; Guangdong province	327	6-9y	Culture	39	6
Luo X, 2006 [22]	2003	Zhongshan; Guangdong province	186	3-6y	Culture	45	7
Liu L, 2007 [23]	/	Chongqing	150	/	Culture	15	7
Lai Z, 2007 [24]	/	Dongguan; Guangdong province	546	4-6y	Culture	109	7
Li R, 2007 [25]	2001	Shijiazhuang; Hebei province	890	≤5y	Culture	139	8
Chen D, 2007 [26]	/	Beijing	561	3-6y	Culture	134	6
Wu T, 2007 [27]	2006–2006	Danyang, Binhai; Jiangsu province	656	≤5y	Culture, PCR	389	7
Dong H, 2008 [28]	2005–2005	Shanghai	501	60-92y	Culture	38	7
Wang F, 2009 [29]	2007–2008	Jianggan; Zhejiang province	101	≤5y	Culture, Hi grouping serum, PCR	21	7
Sun J, 2009 [30]	2008–2008	Shenzhen; Guangdong province	380	all ages	Culture, PCR	121	8
Chen H, 2009 [31]	/	Shenzhen; Guangdong province	181	3~6y	Culture	45	6
Ye J, 2009 [32]	2007–2008	Changshan, Dongyang, Jianggan; Zhejiang province	301	≤5y	Culture, Hi grouping serum	43	7
Meng X, 2009 [33]	/	Shenzhen; Guangdong province	360	3-7y	Culture, Hi grouping serum	90	7
Zhang J. 2010 [34]	2009	Quzhou; Zhejiang province	70	3-5y	Culture, Hi grouping serum	12	6
Li Y, 2010 [35]	2008	Dongyang; Zhejiang province	238	≥6 m	Culture, Hi grouping serum	21	6
Wang J, 2010 [36]	2007–2009	Weihai; Shandong province	820	/	Culture	98	6
Zhang L, 2011 [37]	/	Dongguan; Guangdong province	600	12-18 m	Culture	25	7
Di M, 2012 [38]	2010–2011	Dongcheng; Beijing	834	1–12y	Culture, Hi grouping serum	73	7
Ye Y, 2012 [39]	/	Jinshan; Shanghai	390 children & 240 elders	≤14y & 60-80y	Culture	82	7
Ping G, 2013 [40]	2009–2010	Xuanwu; Beijing	600	12-18 m	Culture	27	7
Zhai R, 2013 [41]	/	/	158	20-34y	Culture	8	6
Qiao H, 2013 [42]	2010	Zhangjiakou; Hebei province	100	3-5y	Culture, PCR	31	6
Chen X, 2013 [43]	/	/	400	/	Culture	125	6
Wang Y, 2013 [44]	/	Baoding; Hebei province	162	all ages	Culture, Real-time PCR	62	8
Xiong W, 2014 [45]	2013	Chongqing	150	≤6y	Culture	11	6
Zhai R, 2014 [46]	/	/	70	17-32y	Culture	11	6
Zhao X, 2015 [5]	2012–2013	Huairou; Beijing	472	2–5y	Culture	45	6
Pan H, 2016 [4]	2010–2011	Chaoshan; Guangdong province	1088	2-6y	Culture	20	8

/: The information were not described. PCR: Polymerase chain reaction

### Overall pooled pharyngeal carriage of *H. influenzae* among healthy population

**Fig. 2:**
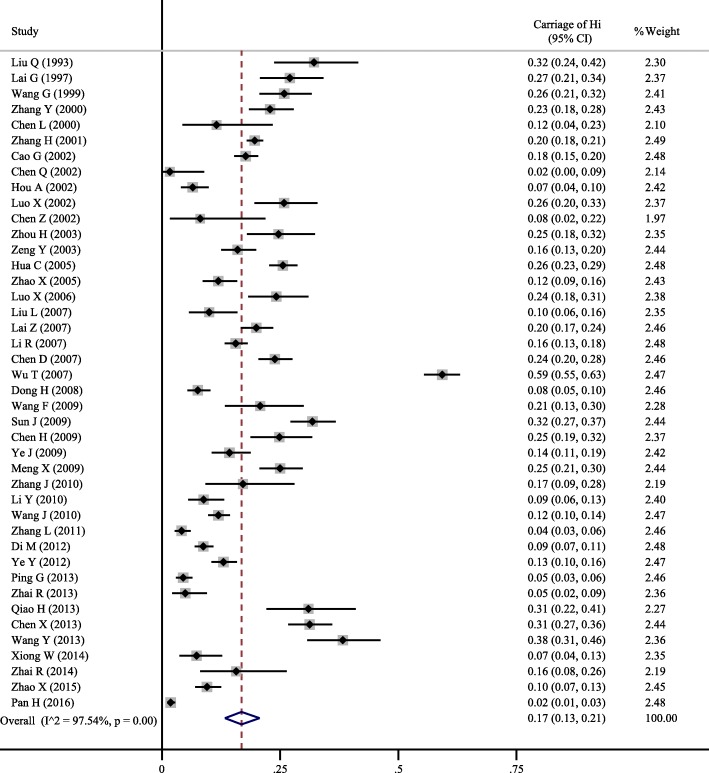
The overall pooled carriage proportion of Hi (*H. influenzae*) among healthy population

A total of 42 studies [4, 5, 7–46] including 17,388 participants reported the carriage proportion of *H. influenzae*, and the overall pooled proportion of carriage was 0.17 (95% *CI*: 0.13–0.21) with significant between-study heterogeneity (*I*^2^ = 97.54%, *p* < 0.01) (Fig. 2; Additional file 1).

**Fig. 3:**
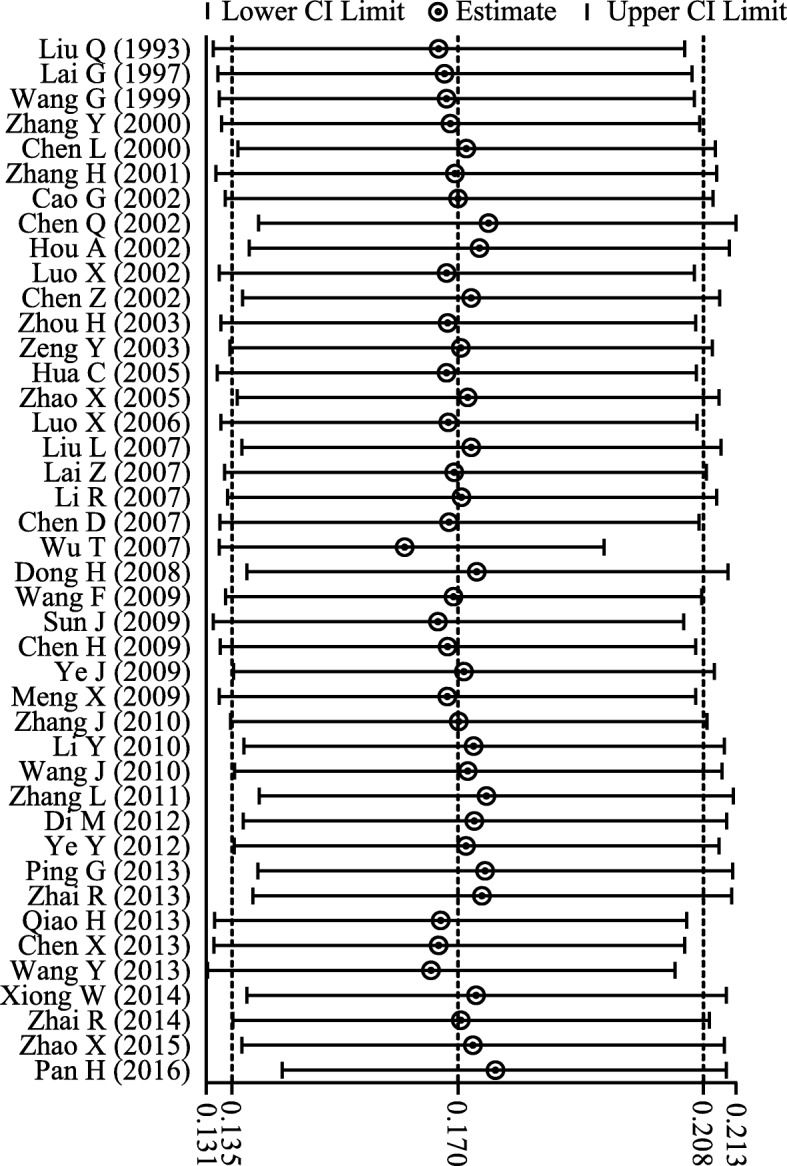
Sensitivity analysis for the effect of individual studies (given named study in the Y axis is omitted) on the pooled carriage proportion

The result of sensitivity analysis showed that the pooled carriage proportion varied from 0.161 (0.133–0.193) (When Wu [27] was excluded) to 0.176 (0.143–0.212) (When Pan et al. [4] was excluded), and indicated that the stability of overall carriage proportion of *H. influenzae* was not influenced by a single study (Fig. 3; Additional file 2).

**Fig. 4:**
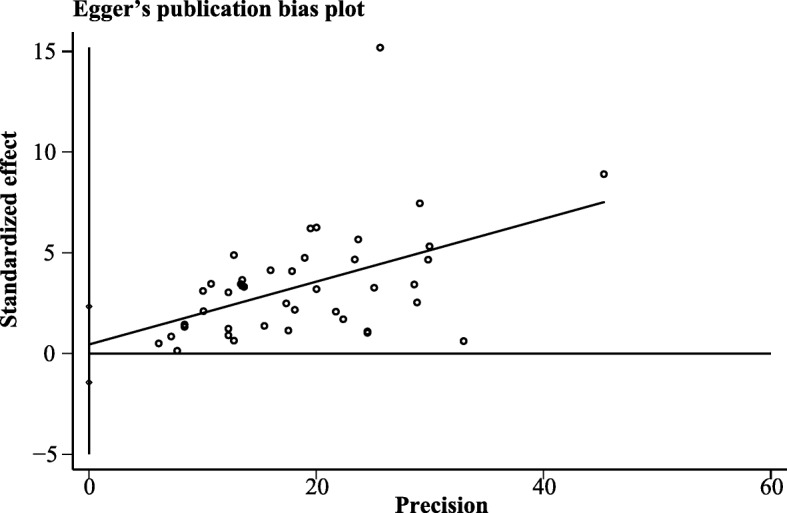
The Egger’s funnel plot of the 42 included studies in the meta-analysis

Egger’s publication bias plot was generated, and the visual symmetry of the funnel plot suggested that there was minimal publication bias (Fig. 4). Moreover, both of the results of Egger’s test (*p* = 0.63) and Begg’s test (*p* = 0.46) also indicated that there was mimimal potential risk of publication bias.

### Subgroup analyses

Among the 42 studies [4, 5, 7–46], 31 studies [4, 5, 7–9, 11–15, 17–27, 29, 31–34, 37, 38, 40, 42, 45] examined children and 5 studies [16, 28, 41, 43, 46] examined adults. Two studies [10, 39], that included both children and adults, were separated at 18 years old and then split into the different subgroups. The rest of 4 studies [30, 35, 36, 44] were not included in the subgroup analysis because of the insufficient age information of participants. The pooled carriage proportion of *H. influenzae* was 0.17 (0.13–0.21) for children (*n* = 14,137), and 0.14 (0.07–0.23) for adults (*n* = 1651). There was no significant heterogeneity between subgroups by age (*p* = 0.65), while significant heterogeneity within subgroups were found (Children: *I*^*2*^ = 97.79%, *p* < 0.01; Adult: *I*^*2*^ = 95.26%, *p* < 0.01).

A total of 12 studies [5, 7, 13, 21, 22, 25–27, 30, 31, 39, 44] described the gender-specific carriage proportion of *H. influenzae*, and the pooled carriage proportion was 0.25 (0.16–0.35) for male (*n* = 2506), and 0.24 (0.19–0.31) for female (*n* = 2531), without significant heterogeneity between subgroups (*p* = 0.88). Six studies [7, 12, 33, 37, 38, 40] described the season-specific carriage proportion of *H. influenzae*, and the pooled carriage proportion was 0.10 (0.06–0.17) in spring (*n* = 1077), 0.08 (0.03–0.14) in summer (*n* = 1459), 0.14 (0.11–0.16) in autumn (*n* = 743), and 0.24 (0.11–0.40) in winter (*n* = 1283), without significant heterogeneity between subgroups (*p* = 0.10).

A total of 8 studies (*n* = 1867) [9, 29, 30, 32–35, 44] described the carriage proportion of specific serotypes, and the pooled carriage proportion of NTHi, Hib, and other serotypes (a, c, d, e, and f) were 0.22 (0.13–0.31), 0.01 (0–0.02), and 0 (0–0.01), respectively.

**Fig. 5:**
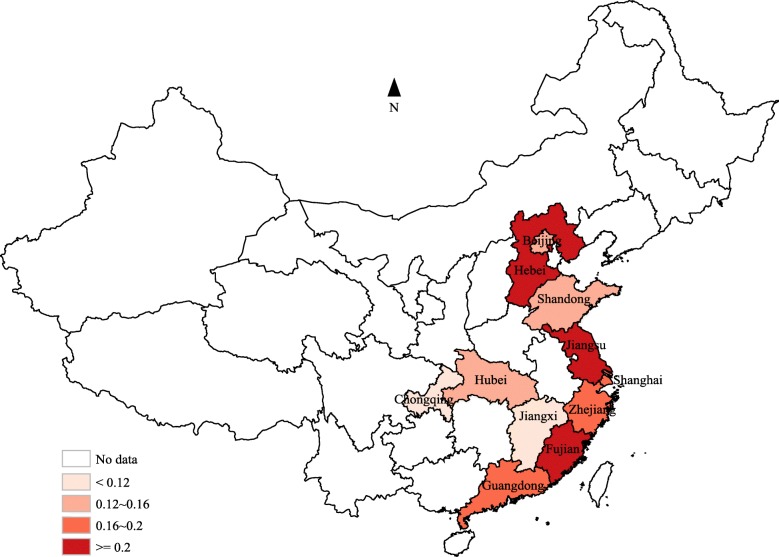
The pooled carriage proportion of *H. influenzae* by province

A total of 39 studies [3, 4, 6–26, 28–40, 42, 44, 45] described their study region, which covered 11 province-level administrative regions. The carriage proportion of *H. influenzae* by provinces varied from 0.02 (0–0.09) in Jiangxi to 0.59% (0.55–0.63) in Jiangsu (Fig. 5; Additional file 3). The heterogeneity between subgroups by province was statistically significant (*p* < 0.01), while the heterogeneity within subgroups were also statistically significant (*I*^*2*^: 88.22%~ 98.19%, all *p* < 0.01).

## Discussion

Globally and in China, most of the studies regarding the carriage of *H. influenzae* were focused on children, which contained substantially different results. For example, in Italy, a study including 717 healthy children aged < 6 years old found that the oropharyngeal carriage proportion of *H. influenzae* was 0.14 [3], while a study of Spain found that the carriage proportion was 0.42 among 900 healthy < 5-year-old children [47], and a study in Belgium indicated the carriage proportion was as high as 0.83 among 333 healthy children [48]. Results of the meta-analysis indicated that the pooled carriage proportion of *H. influenzae* among Chinese healthy children was 0.17 (0.13–0.21), which was a relatively low level. But as presented in Fig. 5, the carriage proportion of *H. influenzae* largely varied by province (0.02–0.59). Since almost all available studies were conducted in limited regions (usually within single city), the differences between provinces has not be previously reported. A possible reason of the variations between provinces might be the insufficient number of studies, which results in unstable results. The available studies only covered 11 in 31 provinces of China, and only single study was available for 5 of those 11 provinces. For 6 provinces with 3 or more studies available, the pooled carriage proportion ranged from 0.11–0.26, which was more stable compared to the range of 11 provinces (0.02–0.59). Moreover, only provinces in the eastern part of China were available. Our results indicated significant between-provinces differences exist, which might influence clinical decision of related diseases according to the actual prevalence of *H. influenzae* in different localities. In particular, no data exists for the western and northern parts of China.

The significant between-study heterogeneity (*I*^2^ = 97.54%, *p* < 0.01), was not explained by the subgroup analysis by age, gender, and season. For age, a possible reason of statistically insignificant between-subgroup heterogeneity was cursory grouping. Because of insufficient age information or inconsistent age grouping in included studies [4, 5, 7–29, 31–34, 37–43, 45, 46], these studies were simply divided into only two subgroups at 18 years old, which might confound the variation of *H. influenzae* carriage proportion with age. For gender, none of the included studies [5, 7, 13, 21, 22, 25–27, 30, 31, 39, 44] reported a statistically significant difference of carriage proportion between males and females, which were consistent with several studies in the United States [49], in Spain [47], and in Italy [3]. It is reasonable to conclude that the gender might not play a role on the carriage of *H. influenzae*. For seasonality, the heterogeneity between subgroups (*p* = 0.10) might be explained by inconsistent sampling time and other confounding factors, since the statistically significant difference between seasons were reported in multiple included studies [12, 33, 38, 40].

The serotype distribution of *H. influenzae* was analyzed in this meta-analysis. Different from the countries which introduced the Hib vaccines in which the Hib was hardly detected [3, 47, 49, 50], results of the present meta-analysis indicated that the carriage proportion of Hib among Chinese healthy population was 0.01, which was lower than a previous meta-analysis study indicating the carriage proportion of Hib for healthy children in China was 0.06 [51]. The reason for the lower carriage proportion in our study could be that we excluded studies that only reported the carriage proportion of Hib. The including of Hib vaccines into the national immunization program should be considered in China, or at least, in some provinces with higher carriage proportion of Hib. Our results indicated that the carriage proportion of NTHi was 0.22 among Chinese healthy population, which makes it a public health issue worthy of attention. It has been reported that the NTHi was frequently identified as the pathogenic bacteria for otitis media, pneumonia, sinusitis [1, 52].

There were some limitations in this meta-analysis. First, the difference of precision of inspection, a possible important reason which influence the detection results of carriage of *H. influenzae*, was unable to be analyzed in this meta-analysis. The precision of inspection would be affected by various factors including the way of swabbing, the time until culturing, the laboratory conditions, the operation of staff, storage and transportation of specimen, and others. These factors were unable to be collected from included articles. Secondly, the meta-regression was not performed because of the needed information was unavailable.

## Conclusion

In China, the pharyngeal carriage proportion of *H. influenzae* among healthy population was at a relatively low level, but it largely varied by study region. Therefore, well-designed nationwide survey with uniform sampling and inspection method on the carriage proportion of *H. influenzae* among healthy population, especially in children, is urgently needed, to clarify the actual prevalence of *H. influenzae* in China, which will contribute to the decision on the prevention and control strategies of related diseases.

## Additional files

Additional file 1: Data collection form and the result of collection. (XLSX 24 kb)

Additional file 2: Result of sensitivity analysis. (XLSX 14 kb)

Additional file 3: The pooled carriage proportion of *H. influenzae* by province. (XLSX 8 kb)

## Data Availability

We declare that the data supporting the conclusions of this article are fully described within the article.

## Abbreviations

H.: *Haemophilus*
Hib: *Haemophilus influenzae* type B
NTHi: Non-typeable *Haemophilus Influenza*
STROBE: Strengthening the Reporting of Observational Studies in Epidemiology
